# Collection of cancer Patient Reported Outcome Measures (PROMS) to link with primary and secondary electronic care records to understand and improve long term cancer outcomes: A protocol paper

**DOI:** 10.1371/journal.pone.0266804

**Published:** 2022-04-15

**Authors:** Elizabeth Stamp, Gemma Clarke, Penny Wright, Galina Velikova, Samantha S. R. Crossfield, Kieran Zucker, Ciarán McInerney, Chris Bojke, Adam Martin, Paul Baxter, Barbara Woroncow, David Wilson, Lorraine Warrington, Kate Absolom, Dermot Burke, Graeme I. Stables, Angana Mitra, Richard Hutson, Adam W. Glaser, Geoff Hall

**Affiliations:** 1 Leeds Institute of Medical Research at St James’s, University of Leeds, Leeds, United Kingdom; 2 School of Sport, Exercise, and Health Sciences, Loughborough University, Loughborough, United Kingdom; 3 Academic Unit of Palliative Care, University of Leeds School of Medicine, Leeds, United Kingdom; 4 Leeds Institute for Data Analytics, University of Leeds, Leeds, United Kingdom; 5 School of Computing, University of Leeds, Leeds, United Kingdom; 6 Academic Unit of Health Economics, School of Medicine, University of Leeds, Leeds, United Kingdom; 7 Leeds Institute of Cardiovascular and Metabolic Medicine, University of Leeds, Leeds, United Kingdom; 8 PPI Member, Leeds Institute of Medical Research at St James’s, University of Leeds, Leeds, United Kingdom; 9 Leeds Teaching Hospitals NHS Trust, Leeds, United Kingdom; University of Technology Sydney, AUSTRALIA

## Abstract

**Introduction:**

More people are living with and beyond a cancer diagnosis. There is limited understanding of the long-term effects of cancer and cancer treatment on quality of life and personal and household finances when compared to people without cancer. In a separate protocol we have proposed to link de-identified data from electronic primary care and hospital records for a large population of cancer survivors and matched controls. In this current protocol, we propose the linkage of Patient Reported Outcomes Measures data to the above data for a subset of this population. The aim of this study is to investigate the full impact of living with and beyond a cancer diagnosis compared to age and gender matched controls. A secondary aim is to test the feasibility of the collection of Patient Reported Outcomes Measures (PROMS) data and the linkage procedures of the PROMs data to electronic health records data.

**Materials and methods:**

This is a cross-sectional study, aiming to recruit participants treated at the Leeds Teaching Hospitals National Health Service Trust. Eligible patients will be cancer survivors at around 5 years post-diagnosis (breast, colorectal and ovarian cancer) and non-cancer patient matched controls attending dermatology out-patient clinics. They will be identified by running a query on the Leeds Teaching Hospitals Trust patient records system. Approximately 6000 patients (2000 cases and 4000 controls) will be invited to participate via post. Participants will be invited to complete PROMs assessing factors such as quality of life and finances, which can be completed on paper or online (surveys includes established instruments, and bespoke instruments (demographics, financial costs). This PROMs data will then be linked to routinely collected de-identified data from patient’s electronic primary care and hospital records.

**Discussion:**

This innovative work aims to create a truly ‘comprehensive patient record’ to provide a broad picture of what happens to cancer patients across their cancer pathway, and the long-term impact of cancer treatment. Comparisons can be made between the cases and controls, to identify the aspects of life that has had the greatest impact following a cancer diagnosis. The feasibility of linking PROMs data to electronic health records can also be assessed. This work can inform future support offered to people living with and beyond a cancer diagnosis, clinical practice, and future research methodologies.

## Introduction

### Cancer survivorship

Improvements in cancer screening, early detection, and treatments mean there are an estimated 2.5 million people living with and beyond cancer in the United Kingdom, which is expected to rise to 4 million by 2030 [1]. Cancer is increasingly viewed as a chronic condition [2, 3], with median overall survival in cancer patients increasing since the 1970s [4]. Though improvements in treatments are reducing mortality and extending life, cancer continues to impact upon people’s lives long after treatment is over. Cancer survivorship refers to the time of diagnosis until the end of life and focuses on aspects of life such as physical, mental, emotional, social, and financial implications that occur from diagnosis, throughout treatment, and beyond [5]. Cancer survivors can face profound and lasting, physical, social, financial and psychological complications secondary to cancer diagnosis and treatment, with an estimated 25% of cancer survivors facing poor health or disability [6, 7].

Cancer survivorship has moved from being marginal to being mainstream on the health agenda [8–10], with survivorship care now perceived as a societal, third sector, and government priority [11]. Aftercare is increasingly provided through risk-stratified pathways, as hospital follow-up is being reduced and more care is being delivered within primary care [8, 10]. A primary care electronic health record (EHR) study reported an increased incidence of chronic illnesses, such as heart failure, coronary artery disease, dementia and diabetes mellitus were identified among survivors of different types of cancers, as compared to matched controls [12]. However, there is currently little evidence concerning: (i) the magnitude and variability between patients in terms of late morbidity, (ii) the costs for primary care, hospitals and patients associated with life after primary cancer treatment, (iii) the cost effectiveness of aftercare services, or (iv) the requirements of users of these services [11].

### Patient Reported Outcome Measures (PROMs)

Healthcare providers strongly focus on the clinical outcomes and tend to give limited consideration to symptom intensity, particularly with subjective symptoms such as fatigue and dyspnoea [13–15]. The importance of capturing the patients’ own perspectives is becoming recognised and incorporated into clinical practice and research [16–18]. PROMs (Patient Reported Outcome Measures) are validated questionnaires completed directly by patients on their health and symptoms. In the UK, the National Health Service (NHS) White Paper, *Equity and Excellence* [19], recommends the wider use of PROMs and patient experience data to place patients at the heart of the health service. PROMs studies have revealed the significant unmet needs of cancer survivors, for example: for breast cancer, studies have shown deficits in physical and social functioning, arm and/or shoulder pain, fatigue, sexual difficulties, insomnia and financial issues [20–25]; for colorectal cancer survivors, studies have indicated bowel and/or urinary control issues, constipation, diarrhoea, sexual difficulties, fatigue, pain [26–28]; and within ovarian cancer, sexual dysfunction and psychosocial issues such as distress depression and anxiety [29–31].

Conversely, some studies have found that for certain cancers, survivors score similarly to the general population for some aspects of quality of life [22, 28, 31–33]. However, there remains a lack of high-quality large-scale studies comparing the quality of life (QoL) for long-term cancer survivors with age and gender matched controls and larger sample sizes. The department of Health recommended the introduction of an innovative quality of life metric to track and respond to the long-term impact of cancer. The Independent Cancer Taskforce in their strategy for England 2015–2020 [9] and NHS Long Term Plan [34] have recommended the introduction and implementation of Quality of Life data collection for cancer survivors in England. Pilot projects are underway looking at electronic methods of patient self-reported questionnaires. Therefore, this paper proposes a protocol to test the feasibility of integrating PROMs data into NHS systems in order to link with PROMs and healthcare records, and to gain a broader picture of life following a cancer diagnosis compared to matched controls.

### Comprehensive Patient Records (CPR)

The study outlined in this paper is part of a larger programme of work called C*omprehensive Patient Records for Cancer Outcomes* (known as CPR) [35, 36]. CPR aims to develop a clearer picture of what happens to people living with and beyond cancer, and the costs to individuals and the healthcare system associated with their care, by securely linking de-identified information from patient questionnaires, electronic primary care and hospital records. The analysis is based upon data drawn from the records of 100,000 cancer patients and 500,000 matched controls. The CPR study comprises five workstreams: (1) data linkage, (2) co-morbidity and late effects of cancer, (3) identifying and predicting recurrence, (4) a health economic analysis of the long term costs of cancer to individuals and the healthcare system, and (5) patient reported costs of living with and beyond cancer (known as the Patient Reported Outcome Measures (PROMs) workstream). The PROMs workstream is the focus of this paper.

CPR PROMs involves collecting PROMs data directly from a subset of the main study population, and integrating it with an identical duplicate of the data collected from Electronic Health Records (EHR) in the main CPR study for this subset of patients. In doing so, CPR PROMs will test the feasibility of collecting PROMs from patients, and linking the PROMs data to patient records. This will enable a truly ‘comprehensive patient record’ to provide a broad picture of what happens to cancer patients across their cancer pathway, and the long-term impact of cancer treatment. Comparisons can be made between the cases and controls, to identify the aspects of life that has had the greatest impact following a cancer diagnosis.

### Aim

The primary aim of this CPR PROMs study is to examine the long-term QoL, personal finances, psychological health, physical health, and social impacts of people living with and beyond a cancer diagnosis. The secondary aim of the study is to test the feasibility of the collection of PROMs data and the linkage procedures of the PROMs data to routine data, to investigate the full impact of cancer on individuals across a wide range of domains. Linkage of the PROMs data to routine data could enable a truly ‘comprehensive patient record’ to be created showing the long-term effects of cancer on factors such as quality of life, health, co-morbidities, and well-being.

### Objectives

1. To collect PROMs data from a cohort of cancer survivors in breast, colorectal and ovarian cancer, and compare these with PROMs data collected from matched individuals without cancer. Data will include: long-term QoL, personal finances, psychological, and social impacts.
2. To integrate PROMs data with de-identified routinely collected primary care and hospital records data using the secure systems for data linkage approved for our wider programme of work [35, 36].
3. To test the feasibility of the PROMs collection and data linkage procedures for use in other research studies and patient care.

**Fig 1:**
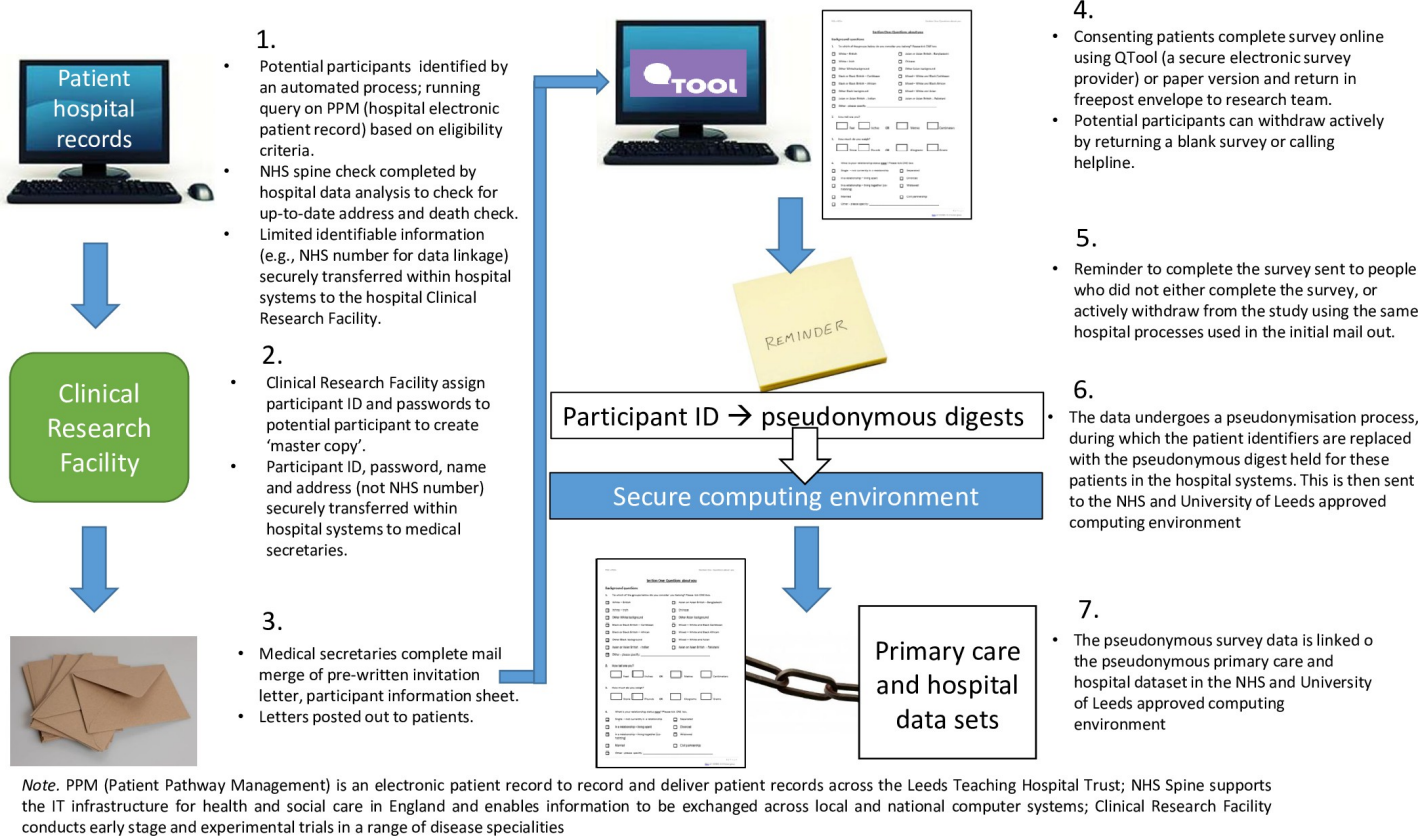
Diagram illustrating a brief over view of CPR PROMs data collection and pseudonymisation. *Note*. PPM (Patient Pathway Management) is an electronic patient record to record and deliver patient records across the Leeds Teaching Hospital Trust NHS Spine supports the IT infrastructure for health and social care in England and enables information to be exchanged across local and national computer systems Clinical Research Facility conducts early stage and experimental trials in a range of disease specialities.

An overview of the data collection procedures and pseudonymisation is displayed in Fig 1.

## Materials and methods

### Study design

The study uses a cross-section of matched-control PROMs survey with data linkage to de-identified hospital data (via Leeds Teaching Hospitals NHS Trust, LTHT) and primary care (via The Phoenix Partnership, TPP).

### Study setting

Patients will be recruited from LTHT, one of the largest teaching hospitals in Europe. The hospital provides local and specialist services for the immediate population of 770,000 and regional specialist cancer care for up to 5.4 million people. TPP provide ResearchOne as a primary care research database containing de-identified data extracted from consenting SystmOne GP practices. Some primary care practices using SystmOne have agreed for their practice data to be included in a de-identified format on ResearchOne (a health and care research database which contains linked data from primary and secondary health care), which is also provided by TPP. Full details of the health records linkage is provided in the protocol for our wider programme of work [35, 36].

### Participants

Patients will be eligible for this study if they are registered on the LTHT electronic patient record and listed as using a Leeds Clinical Commissioning Group primary care practice. Specific inclusion and exclusion criteria for the cases and matched controls are specified below.

### Inclusion criteria for cancer cohort

1. A diagnosis of colorectal cancer (ICD-10 C18—C20), breast cancer (ICD-10 C50) or ovarian cancer (ICD-10 C56)
2. Approximately five years beyond their first cancer diagnosis (e.g., index date = 2013 ± 4 years). Date. Index date was the patient’s clinic appointment date. The year band is expanded incrementally until the full cancer cohort is obtained.
3. 18–100 years old on date of diagnosis

### Inclusion criteria for control cohort

1. Reviewed by LTHT dermatology services on a two week wait for potential skin cancer, but were not diagnosed with cancer
2. No cancer diagnosis recorded within LTHT records at any time.
3. One dermatology appointment within 12 month of the matched case’s cancer diagnosis (their index date).
4. Age 18–100 on appointment date.
5. No further dermatology appointment in the 12 months following their index date.
6. Could be matched to cases by age, sex and primary care practice.

### Sample size

The survey will be sent out to around 6,000 potential respondents. The estimated consent rate for cases is around 60% which is based on similar postal PROMs surveys recruitment rates of: 63% [37], 68% [38], 49.1% [39]. The anticipated consent rate for controls is lower and estimated at between 23.9% [39] and 30% based on recent returns from a control study matched to cases of men with prostate cancer [40]. Based on expected consent rates, this should result in a sample size of around 1,000 to 1,200 in each group (cases, controls). A power sample size calculation was undertaken using the study’s main measure the QLACS (Quality of Life in Adult Cancer Survivors), and this number exceeds the number of respondents required for 90% power for all the data analysis subsets.

#### Matching process

Cancer cases to control cases will be at a ratio of one to two (2,000 controls and 4,000 cases).

Match the cancer case (2:1 ratio of controls to cancer cases)

- Sex.
- By primary care practice.
- Age: date of birth is within (+/-) 30 months. The time band is expanded incrementally until the PROMs cancer cohort has two matches. If an expansion of the band is necessary, the matching process will be re-run for the whole PROMs cohort to avoid bias from some cases being closer matched than others (other than at random).

### Exclusion criteria cases and controls

All patients must have a valid NHS number. Patients will be excluded if they are on the national opt out register, are identified by LTHT clinicians as not meeting the eligibility criteria (e.g., at risk of lacking decision-making capacity), or if patients have informed LTHT they want to opt out of research. The primary care data available from the main CPR study may not be available for all patients within the CPR PROMs cohort. This is due to some patients who have opted out of R1 (an electronic database which stores non-identifiable patient records that have ethical approval to be used in research) being excluded from the main study dataset and some patients not attending a practice that uses R1.Thus, primary care data will only be linked where it is eligible and available.

### Identification

Potential participants will be identified from LTHT’s internal computer patient management system, Patient Pathway Manager using a computerised search query created from the inclusion and exclusion criteria. If the query search produces too many results, potential participants will be randomly selected using a randomisation process. We will check the NHS Spine immediately prior to the mail out to ensure surveys are not mailed out to patients who have been recorded as deceased, and that ‘up to date’ names and addresses are retrieved.

### Survey and invitation letter design

The survey content comprises psychometrically sound questionnaires relevant to the cancer survivor groups under investigation. The content was informed by the results of extensive engagement with patients and clinical staff. This included: clinical shadowing in consultant-led and nurse-led follow-up clinics for breast, colorectal and ovarian cancer; conversations with cancer clinicians and specialists; a ‘word wall’ activity in patient clinics to allow cancer patients to rank and discuss issues of importance to them (the ‘word wall’ is a novel visual method which allows participants to rank items by moving them around a board); and an electronic online ‘word wall’ activity undertaken by active service users from the websites Macmillan ‘Cancer Voices’ [41] and ‘use MY data’ [42, 43]. The survey was limited to be less than 120 items as a recent PROMs survey of cancer survivors had a response rate of 60% with 120 items [37]. A final review by patients and staff was undertaken to ensure the full scope of topics was included without being too burdensome. The survey takes around 30 minutes to complete. Patient and public involvement was also utilised to inform the letter that was sent out with the survey to invite people to participate in the study. People who have not been diagnosed with cancer provided feedback on receiving a letter about an oncology study and minimising anxiety.

### Survey measures

The survey comprises four sections with 108 items for cases and 100 items for controls (see Table 1 for overview of survey). The same questions will be used for cancer survivors and the controls unless specific item changes are required for the control group.

**Table 1:** An overview of the PROMs included in the survey.

Survey section	Questionnaire title and brief description	Item information/response format and scoring
Section 1: Questions about you	**Demographics**. Seven questions on participants demographics (e.g., ethnicity, height, weight, relationship status, education, health conditions)	N/A
Section 2: Questions about your health	**EQ-5D-3L and Visual Analogue Scale (VAS**) [44]. 6 item scale measuring respondent health ‘today’. Subscales include mobility, self-care, usual activities, pain, anxiety/depression. Visual analogue scale provides a single index value for health status	5 items rated from 1 (no problems) to 3 (severe problems). Visual analogue scale measured from 0 (worst imaginable health state) to 100 (best imaginable health state)
	**Quality of Life in Adult Cancer Survivors (QLACS)** [45]. 47 item questionnaire to assess Health related quality of life in cancer survivors and non-cancer populations. Scores five cancer-specific domains (financial problems, distress family, distress recurrence, appearance problems, benefits) subscales, as well as a total score which does not include the benefits subscale. Generic scales scores are physical pain, negative feelings, positive feelings, cognitive problems, sexual problems, social avoidance, fatigue.	Items rated on a 7 point scale from 1 (never) to 7 (always) based on how respondent has felt in last 4 weeks. Scores provided for each domain
	**Cancer survivor identify question** [46]. Measures the extent of adoption of cancer related identify for cases, and perceptions of person living with cancer for control group.	Respondents can select from one of five options: ‘a cancer survivor’, ‘a person who has had cancer’, ‘a cancer patient’, ‘a victim of cancer’, or ‘other’
	**European Organisation for Research and Treatment of Cancer (EORTC)** [47] 14 items from the EORTC question bank used to assess health related functioning and symptoms. Subscales include tingling, muscular pain, urinary frequency, urinary incontinence, urinary symptoms, gastrointestinal symptoms, diarrhoea, constipation, abdominal/Gastrointestinal symptoms	Items rated on a 4 point Likert scale from 1 (*not at all*) to 4 (*very much*). Score provided for each subscale
Section 3: Questions about your lifestyle	Godin Leisure-Time Exercise Questionnaire [48]. 4 item instrument to assess time spent in light, moderate, and strenuous physical activity.	Respondents state how many minutes were spent in light, moderate, and vigorous intensity physical activity in a week. This produces an overall score of ‘active’, ‘moderately active’, and ‘insufficiently active/sedentary’.
	Exercise Benefits and Barriers Scale (EBBS)– 14 item barriers subscale [49]. Subscales measured include family discouragement, exercise milieux, physical discomfort, time expenditure	Exercise barrier items scored on subscale from 1 (strongly disagree) to 4 (strongly agree)
Section 4: Questions about your healthcare and finances	Cancer Care Coordination Questionnaire (CCCQ) [50]. 7 item navigation subscale	Respondents rate statements on a 5-point Likert scale from 1 (never) to 5 (always)
	Financial costs questionnaire. Measures Employment status, social security entitlements, number of days absence from work due to illness (and associated loss of income); care and support received from informal carers; out-of-pocket expenses on prescriptions, hospice, hospital or community health services; travel to and from health and social care appointments; and any other out-of-pocket expenses, use of other health care services funded by the NHS or voluntary and charity sector services	N/A

### Section 1: Questions about you

Patients will be asked to provide basic background information about ethnicity, height and weight, relationship status (at the time of the survey, and at the time of their cancer diagnosis/or 5 years ago for controls), highest level of education, co-morbidities.

### Section 2: Questions about your health

#### EQ-5D-3L

EQ-5D is a standardised measure of health status providing a simple, generic measure of health related quality of life for clinical and economic appraisal [44]. The EQ-5D is the NICE preferred measured for capturing health related QoL [51]. The EQ-5D asks respondents about their health ‘today’, on the day on which they respond to create a profile of their health in the following subscales mobility, self-care, usual activities, pain, anxiety/depression. This profile is then converted to a health related QoL score via a validated algorithm based on UK population stated preferences. The scale is anchored on 1 representing full health and 0 representing the health related quality of life associated with death. Values worse than death are possible and represented by negative scores. In addition, there is a Visual Analogue Scale (EQ-VAS) which records self-rated health on a 0 to 100 scale. The EQ-5D has been used in previous studies with cancer patients, including those with breast, colorectal and ovarian cancer, and has been shown to be reliable and valid in this population [52–56]. Normative population data are available for the 3 level version [57].

#### Quality of Life in Adult Cancer Survivors (QLACS)

QLACS is a 47-item measure of QoL amongst adult survivors of cancer at least five years post diagnosis [45]. The instrument comprises of cancer specific scales and generic scales. The generic scales do not mention cancer and thus make the instrument an appropriate measure to make comparisons between cancer and non-cancer populations [58]. QLACS was highlighted in the 2010 National Cancer Survivor Initiative ‘Vision’ document [17], and has been previously used with breast, colorectal and gynaecological cancer patients [45, 58, 59]. For the cancer specific domains items with the term “cancer” or “cancer treatment” are changed to “health” or “healthcare treatment” within the control group questionnaire. Two subscales are not included in the control group survey as they are deemed as not appropriate: the ‘Benefits of cancer’ and ‘Distress over recurrence”.

#### Cancer survivor identity question

This comprises a single item examining the extent of adoption of four cancer-related identities. The item for the cancer cases group was: ‘Which one of the statements below best describes you/do you relate to most?’ Participants responded selecting one of the following options: ‘ a cancer survivor’, ‘a person who has had cancer’, ‘a cancer patient’, ‘a victim of cancer’, or ‘other’[46]. Similar versions of this question have been used with prostate cancer survivors [60], and with older cancer survivors at least 5 years from diagnosis [61]. This question will be adapted for the control population to relate to their perception of people who have had cancer: ‘Which one of the statements below best describes how you view someone who has had cancer?’

#### European Organisation for Research and Treatment of Cancer (EORTC) Quality of Life Questionnaires

The EORTC questionnaires have been developed for international use to assess health-related QoL in cancer patients [47]. The EORTC has an Item Library of more than 900 individually validated items from over 60 EORTC questionnaires. Its aim is to help users create relevant custom-made item lists specific to their studies. The present study did not use the EORTC QLQ-30 as it was originally developed to cover QoL during cancer treatment as opposed to survivorship. The questionnaires comprise a core generic cancer questionnaire (EORTC QLQ-30), and a number of cancer site-specific modules, which measure health-related functioning and symptoms in a range of scales. The survey included questions from the EORTC item bank, as shown in Box 1.

**Box 1. EORTC subscales included in survey and the EORTC module that they are from**

Tingling/numbness (1 item – Endometrial module)

Muscular pain (1 item – Endometrial module)

Urinary frequency (2 items – Colorectal module)

Urinary incontinence (1 item – Colorectal module)

Urinary symptoms (1 item – Bladder module)

Gastrointestinal symptoms (5 items – Endometrial module)

Diarrhoea (1 item – Core module)

Constipation (1 item – Core module)

Abdominal/Gastrointestinal symptoms (1 item - Ovarian module)

### Section 3: Questions about your lifestyle

#### Godin Leisure-Time Exercise Questionnaire (GLTEQ)

The Godin Leisure-Time Exercise Questionnaire (GLTEQ) [48] assesses leisure time physical activity, and has been used within oncology research [62, 63]. Examples of activities are provided for each intensity category, for this study, examples of exercise have been adapted to the UK cultural context. A scoring algorithm provides an overall exercise score.

#### Exercise Barriers and Benefits Scale (EBBS)

The EBBS is a 43-item summated rating scale consisting of two subscales, Benefits and Barriers [49]. To focus on the barriers to exercise for those living with and beyond cancer, this study uses the ‘barriers’ subscale only; consisting of 14 items. The EBBS was developed for a general population and has been used widely in groups such as the elderly [64–66], and in diseases such as Multiple Sclerosis (MS) [67], cardiovascular disease [68], and cirrhosis [69], but has not been used previously within cancer.

### Section 4: Questions about your healthcare and finances

#### Cancer Care Coordination Questionnaire (CCCQ)

The Cancer Care Coordination Questionnaire (CCCQ) is a recently developed tool to measure patients’ experiences of healthcare coordination, it has two subscales: communication and navigation [50]. This study uses the ‘Navigation’ subscale only; comprising seven items asking ‘how often’ difficulties were experienced over a range of issues such as, making appointments or waiting for test results. The CCCQ has shown to be psychometrically robust in initial testing [50], but has yet to be tested widely.

#### Financial costs questionnaire

The financial costs questionnaire was developed by the health economics team working within the CPR research programme to measure the personal financial impact of cancer. It is an adapted version of the ‘financial cost of cancer’ questionnaire used in the electronic Patient-reported Outcomes from Cancer Survivors study [70].

### Survey administration

The survey may be completed on paper (delivered by post) or online.

### Paper completion

A paper survey with a unique Participant Identification number (PID) and a freepost envelope for return to the research team will be mailed to each potential participant.

### Online completion

The invitation letter will provide a website address for the survey and a unique participant log in (based on the PID) and password. The web-based system (QTool) is hosted securely by the Integrated Research Campus (IRC) at the University of Leeds [71].

### Pilot study

A pilot study of 5% of total research population will be undertaken using the processes detailed below. Should negative responses be received, the study methods and processes will be reviewed and amendments undertaken if required.

### Recruitment

Participant identification and recruitment are undertaken entirely within LTHT, the research team have no access to patient identifiable data (approved by the UK’s Health Research Authority (HRA) Confidentiality Advisory Group (CAG) (Reference: 18/CAG/0119)). The stages are summarised in Fig 1 and outlined in more detail below.

LTHT data analysts will generate a list of potential respondents by creating and running a query on the internal electronic patient management system derived from the eligibility and exclusion criteria. A list of identifiable data (name, address and NHS number) will be extracted and sent to LTHT IT Services using secure LTHT computer systems. A member of the LTHT IT Services team will check the list of potential respondents on the NHS Digital Spine (https://digital.nhs.uk/spine) for name, address and death. The updated list will be sent to the LTHT Clinical Research Facility who will manage the lists. The LTHT Clinical Research Facility conducts early stage and experimental trials in a range of disease specialities.

The clinical Research Facility will allocate PIDs using an automated process and will be the ‘key-holder’ for linking the PID to the individual patient within the secure LTHT systems. An automated ‘mail merge’ process will be used to merge the list of patients’ names and addresses with an invitation letter and survey. The completed ‘mail merge’ templates will be sent to LTHT medical secretaries for printing. Printed letters, signed by a consultant on behalf of the relevant treating Multi-Disciplinary Team (MDT), and containing the Participant Information Sheet will be mailed via the LTHT post room. Following a second NHS Digital spine check, a reminder letter will be sent after four weeks to those who have not responded at all and who have not contacted the research team requesting study withdrawal.

### Consent

Informed consent (ticking or dating the front page of the paper survey or clicking the first page of the online survey) will be sought from participants for taking part in the PROMs survey and for the data linkage to de-identified data from hospital and primary care records, in accordance with the Data Protection Act 2018. The invitation letter and Participant Information Sheet will provide detail of the study aims, methods and processes for participation and withdrawal.

## Data

### PROMs data

All collected PROMs data is non-identifiable and participants are asked not to include any identifiable data on their survey return. Each PROMs survey has a research participant ID. LTHT, who send the invitation and survey, retain a link between the research ID and the patient record (i.e. the PROMs data is reversibly pseudonymous from the perspective of LTHT).

#### Electronic health records data

The EHR data that can be linked to the PROMs data will mirror the data collected in the main CPR study (see S1 Appendix). This includes data such as co-morbidities, appointments, frailty score, and treatment received for diagnosis. All data received will be de-identified. “De-identified data” refers to data that has had all identifiable personal markers removed such as name, address, date of birth and NHS number. De-identified data will come from two data providers:

(1) Hospital data will come from patient hospital records and financial data held to support patient care at LTHT.

(2) Primary care data will come from ResearchOne, where available. ResearchOne is a research database consisting of de-identified data derived from electronic patient records from healthcare providers who use the TPP SystmOne clinical computing system [72]. SystmOne is among the largest clinical databases in the world, containing the electronic records of more than 40 million patients. The data are drawn from a variety of health and care settings across England, including both primary, secondary, and social care providers. Health and social care providers using SystmOne can opt for data to be de-identified and included in the ResearchOne database; individual patients also have the opportunity to opt-out of supplying their de-identified data [73].

### Pseudonymisation and data linkage

LTHT will derive a project-specific pseudonym (called a “digest”) for each patient involved in the project and retain a link from this to the patient record ID. Participants consent to be part of the PROMs study and have their data linked by returning a completed survey. A list of research IDs is derived from the completed surveys and provided to the LTHT data analysts who use this to determine the pseudonymous digest for the PROMs participants. The digests are linked to the non-identifiable PROMs survey data. This is then transferred to a NHS and University of Leeds approved physical or cloud computing environment and linked with hospital data from LTHT and de-identified primary care data from ResearchOne via project specific digests (where the data is available and eligible). The process of linkage, with the linkage field subsequently being destroyed, will mirror the procedure of the main study, so that the research dataset cannot be linked back to data sources. A full explanation of the data linkage and pseudonymisation methodology has been previously published [35, 36]. This process uses the open source software OpenPseudonymiser [74]. The computing systems have advanced computational highly secure infrastructure with accredited certification to the international standard for information security management, ISO/IEC 27001:2013 and Level 2 NHS Information governance (IG) Toolkit.

### Data on non-responders

Invited participants who do not respond to the survey are defined as ‘non-responders’. De-identified double-pseudonymised data will be requested on non-responders for the purpose of testing the response bias of the sample. This will be collected by the process described above, though necessarily without any PROMs data. The Research ID of non-responders is linked with the project-specific digest under the LTHT Fair Processing Notice. The data handling is similarly in accordance with the common law duty of confidentiality because the data is rendered anonymous through de-identification processes. The data providers (LTHT and TPP) have reviewed the data processing (as advised by the Health Research Authority Confidentiality Advisory Group) as part of the main study: C*omprehensive Patient Records (CPR) for Cancer Outcomes*, Research Ethics Committee reference: 16/NE/0155), and deemed the data to be de-identified. It is under the data providers’ approval that this data is used.

### Data input

For those participants choosing to complete online, the weblink provided will allow participants to input their data directly onto QTool. For those choosing to return the paper survey, the surveys will be input onto QTool by LTHT medical and university secretarial staff.

### Data storage

#### Electronic non-identifiable survey data storage

Survey data will be stored and managed initially on secure University of Leeds servers. The data (with PIDs) will be transferred to the Clinical Research Facility for secure linkage to LTHT project-specific digests and then transferred using the methods described above.

#### Paper survey storage

The returned hard copy paper questionnaires will be securely stored in locked filing cabinets in the PROMs research team office. The paper questionnaires will be destroyed at the end of the study, after the data have been uploaded and validated.

#### Linked de-identified data storage

The linked de-identified data will be held in an NHS and University of Leeds approved physical or cloud computing environment. A number of cloud computing services have undergone detailed analysis and review by the Leeds Teaching Hospitals NHS Trust information governance team. This process included a detailed analysis of the security arrangements relating to the storing and access of data in addition to reviews of the external accreditation that has been provided such as NHS Digital Data Security and Protection Toolkit certification and international organisation for standardisation 27001 accreditation [75].

#### Long-term data storage and data sharing

Identifiable personal data will be destroyed at the end of the study. De-identified data will be stored for 10 years. Derived (aggregated) PROMS survey datasets may be made available for data sharing to underpin and validate research findings. The IRC Data Services Team will assess datasets for disclosure, and release these for storage in an open repository such as the Research Data Leeds Repository. They will be given a Digital Object Identifier (DOI).

## Ethical issues

### Compliance and approvals

This study will be conducted in accordance with both the Data Protection Act (2018) and the General Data Protection Regulation 2018. All clinical staff and research staff involved in the study have NHS (or honorary NHS) contracts and work to NHS codes of practice concerning confidentiality, information security management and records management (NHS Code of Confidentiality, 2003). This project was approved by the HRA (04/10/2018 IRAS Reference: 233983); HRA NHS Research Ethics Committee (10/09/2018 Research Ethics Committee Reference: 18/LO/1043); HRA NHS Confidentiality Advisory Group (CAG) approval (02/08/2018 CAG Reference: 18/CAG/0119); LTHT Capability and Capacity approval (14/03/2018); and received NIHR Portfolio study (12/09/2018 Reference: CPMS 39154).

## Data analysis

Response bias will be examined using the de-identified data from the non-responders. Descriptive statistics will be used to describe the participation characteristics and the PROMS outcome measures. Inferential statistics will explore the differences between the cancer cases and matched controls. Missing item-level data will be reported and checked for systematic biases. Where possible, and subject to questionnaire guidance, missing values may be imputed using multiple imputation techniques.

### Primary outcome

The QLACS is the primary outcome measure. The generic summary scores of the cancer survivor’s cohort will be compared to those of the matched controls to identify statistically and clinically significant differences. Comparisons will also be made on the individual domains of the QLACS, excluding those where comparative data is not available for controls (distress-recurrence and benefits of cancer). Subgroup analysis will be undertaken by cancer type.

### Secondary outcomes

Secondary measures will be used to further explore the differences between the cancer survivors and the matched controls using the PROMs measure and the linked primary and secondary data, on the topics of:

- Health and quality of life using the EQ-5D-3L and VAS, and items from the EORTC bank.
- Difficulties navigating the healthcare system, using the PROMs measure CCCQ (Cancer Care Coordination Questionnaire) and de-identified healthcare appointment and co-morbidities data from primary and secondary care;
- Exercise, using the PROMs measures EBBS (Exercise Barriers and Benefits Scale) and GLTEQ (Godin Leisure-Time Exercise Questionnaire);
- Cancer survivor identity, using the included question on identity;
- Financial costs of cancer and personal financial impact, using the data collected in the questionnaire

### Feasibility assessment

#### Feasibility assessment is the secondary aim of this protocol

Feasibility of the collection of PROMs and linkage to EHRs will be assessed at the end of the study by the research team. The key outcome for testing feasibility will be the response rate (defined as the participant consenting and returning data). Based on previous research, our desired response rate is at least 50% for cases and 30% for controls [37, 38]. These rates have taken into account the challenge of recruiting participants who are five years after diagnosis or have not been diagnosed with cancer. The research team will also meet to discuss: feedback offered from participants, method of completion (online versus paper), the representativeness of the sample, quality of collected PROMs data, ease of using systems, and costs.

## Discussion

PROMs have been reported to have the potential to transform healthcare by enabling patients and clinicians to make better decisions, and by allowing comparisons of different services to stimulate improvements [18]. However, a number of significant challenges were identified that need to be overcome to maximise their contribution, including; minimising costs, achieving high participation, providing appropriate outputs and overcoming methodological challenges [18]. CPR PROMs seeks to address these challenges. To our knowledge, CPR PROMs is the largest scale study worldwide to date to link non-identifiable PROMs data from long-term cancer survivors with de-identified primary and secondary data for cancer survivors and age and gender matched controls, and the first to do so utilising routine NHS systems. The findings from CPR PROMs will go some way to overcoming the challenges for routine PROMs use, as previously outlined [18]. The findings will provide detailed data on cancer survivors’ health and quality of life outcomes which may inform improvements in service provision, improved information for patients and clinical teams, optimising post-treatment support, and informing future research.

This study will test the feasibility and acceptability of collecting PROMs data within an NHS system (i.e., data entered onto QTOOL), using no external survey provider or other external organisations. As such it is a real test of the feasibility of collecting PROMs data within such settings. This is of particular importance because the Independent Cancer Taskforce in their strategy for England 2015–2020 [9] and the NHS Long Term Plan [34] both recommended the introduction and implementation of Quality of Life data collection for cancer survivors in England. The methods trialled during this study will indicate the feasibility of adopting such processes into routine care.

This study has involved considerable input from patient and public involvement and Engagement and clinical staff in the study design and consideration of ethical issues. Active service user input on document content and style, such as the invitation letter and participant information sheet was of crucial importance. A separate publication on the patient and clinician engagement in the study design is forthcoming.

## Strengths and limitations

A strength of the current protocol is that it is, to our knowledge, the largest scale study to link non-identifiable PROMs data from long-term cancer survivors with de-identified primary and secondary data for cancer survivors and age and gender matched controls. It does so utilising routine NHS systems. This demonstrates the potential for future research to take a more holistic approach through incorporating PROMs data with EHRs (e.g., appointments, treatment, co-morbidities), as well as providing insights for healthcare decision making. A further strength of this study is that all processes are conducted within the NHS without the use of external providers.

One limitation of the current protocol is that the data linkage of routine health records and PROMs can only take place for patients who are part of an R1 surgery and have not opted out of sharing their data.

## Supporting information

S1 Appendix Data list from the main study. (PDF)

## Abbreviations

CAG: Confidentiality Advisory Group
CCCQ: Cancer Care Coordination Questionnaire
CPR: Comprehensive Patient Records
EBBS: Exercise Barriers and Benefits Scale
EORTC: European Organisation for Research and Treatment of Cancer
EQ-5D: EuroQol Group 5 Dimensions of Health Questionnaire
GLTPEQ: Godin Leisure-Time Exercise Questionnaire
HRA: Health Research Authority
IT: Information Technology
LTHT: Leeds Teaching Hospitals NHS Trust
NHS: National Health Service
PID: Patient Identification
PROMs: Patient Reported Outcome Measures
QLACS: Quality of Life in Adult Cancer Survivors
QoL: Quality of Life
REC: Research Ethics Committee
TPP: The Phoenix Partnership
