# Supplementary material for: Collection of cancer Patient Reported Outcome Measures (PROMS) to link with primary and secondary electronic care records to understand and improve long term cancer outcomes: A protocol paper
Source: PLoS One. 2022 Apr 15;17(4):e0266804. doi: 10.1371/journal.pone.0266804 (PMC9012381; doi:10.1371/journal.pone.0266804)
Supplement: S1 Appendix — (PDF) [file pone.0266804.s001.pdf]

Comprehensive Patient Records for Cancer Outcomes

Data List

Table of Contents

1. Data Items accessed by the research team, from TPP's SystmOne derived ResearchOne database 1

2. Data Items accessed by the research team, from LTHT's PPM and financial data derived data warehouse 4

3. Data handling by the IRC Data Services Team 8

Data Items Highlighted in Bold Represent Changes from Previous Data List (V3.0)

1. Data Items accessed by the research team, from TPP's SystmOne derived ResearchOne database

1. Demographics including
    a. age (<1 years and 1-4 years and 5-year bands thereafter, to 80+)
    b. Rurality score
    c. IMD quintile
    d. CCG
    e. sex
    f. ethnicity
2. Date last seen by member of primary care team (GP, practice nurse etc.), date of last repeat prescription.
3. **TPP's derived Frailty scores:**
    a. **eFrailty and other frailty scores 5 years prior to cancer diagnosis or control patient hospital contact event.**
    b. **Month, year and new score for subsequent changes in Frailty score**
4. Coding re cancer diagnosis (including primary and secondary cancer and recurrences) and/or two week wait or other urgent suspected cancer referral or control patient hospital contact event including
    a. National standard coded data, e.g. READ codes
    b. Stage at presentation
    c. Morphology
    d. Grade
    e. Molecular phenotype e.g. ER/PR, HER2, EGF

5. The presence/absence of specific co-morbidities (based largely on ACE-27, the 27-item comorbidity index for patients with cancer (HSCIC data dictionary, 2016) at or prior to diagnosis or control patient hospital contact event including:
    a. Myocardial Infarction
    b. Angina / Coronary Artery Disease
    c. Congestive Heart Failure
    d. Cardiac arrhythmia
    e. Peripheral Arterial Disease
    f. Hypertension
    g. Venous disease including DVT, PE
        i. Venous Thromboembolic Disease
        ii. Venous Insufficiency
        iii. Varicose veins
    h. Restrictive lung disease or COPD
    i. Asthma
    j. Liver Disease including chronic hepatitis
    k. Stomach / Intestine including ulcers, malabsorption and inflammatory bowel disease
        i. Peptic Ulcer Disease
        ii. Malabsorption
        iii. Inflammatory Bowel Disease
    l. Pancreatitis
    m. Renal Disease
        i. Grade 1
        ii. Grade 2
        iii. Grade 3
        iv. Grade 4
        v. Grade 5 (End Stage)
    n. Diabetes mellitus
        i. Type 1
        ii. Type 2
        iii. Other or Unspecified
        iv. Insulin dependent
        v. Diabetes Medication excluding insulin
        vi. No Medication
    o. Cerebral Vascular Accident
        i. Stroke
        ii. TIA
    p. Paralysis including hemiplegia, paraplegia
        i. Paralysis
        ii. Cord injury and myelopathy
    q. Neuromuscular
        i. Demyelinating disease
        ii. Parkinson's Disease
        iii. Motor Neurone Disease
    r. Dementia
    s. Mental Health Disorders
        i. Anxiety
        ii. Depression
        iii. Substance abuse including alcohol and illicit drug use
        iv. Post-Traumatic Stress Disorder
        v. Mania/Bipolar
        vi. Behavioural Disorder
        vii. Schizophrenia / Psychosis

viii. Perinatal Mental Health Disorder
ix. Obsessive Compulsive disorder
x. Somatisation / Dissociation
xi. Eating Disorder
xii. Attention Deficit and hyperactivity Disorder

t. Rheumatologic including rheumatoid, connective tissue disorder
   i. Rheumatoid Arthritis
   ii. Psoriatic Arthritis
   iii. Ankylosing Spondylitis
   iv. Gout
   v. Back pain
u. HIV status
v. Obesity
w. Hyperlipidaemia
x. Women's Health Outcome measures
   i. Assisted delivery
   ii. Caesarean Section
   iii. Cerebral venous sinus thrombosis
   iv. Cervical dysplasia
   v. Ectopic pregnancy
   vi. Genital prolapse
   vii. Infertility
   viii. Maternal death
   ix. Menopause
   x. Menstrual Disorders
   xi. Miscarriage
   xii. Molar pregnancy
   xiii. Multiple pregnancy
   xiv. Normal delivery
   xv. Painful intercourse
   xvi. Post-natal depression
   xvii. Precocious puberty
   xviii. Pregnancy
   xix. Psychosexual dysfunction
   xx. Sterilisation
   xxi. Stillbirth
   xxii. Urogenital Fistula
   xxiii. Combined oral contraception
   xxiv. Contraceptive diaphragm
   xxv. Contraceptive implant
   xxvi. Contraceptive injectable
   xxvii. Contraceptive patch
   xxviii. Emergency Hormonal Contraception
   xxix. IUD
   xxx. IUD/IUS Removal
   xxxi. IUS
   xxxii. Progestogen only oral contraception
   xxxiii. Removal of Contraceptive Implant

6. Time since original diagnosis of specific co-morbidities and categories (as above) measured in 3 monthly increments in the year prior to cancer diagnosis (or control patient hospital contact event) and annually if longer than one year prior to diagnosis.
7. Development of specific co-morbidities (as above) annually for 10 years and 5-yearly intervals thereafter following cancer diagnosis/control patient hospital contact event.
8. Use of out of hospital and community care resource over time, e.g. number of face-to-face, telephone and home visit by members of the primary care team, blood tests, scans (CT, bone, MRI), monthly for 10 years following cancer diagnosis/control patient hospital contact event.
9. Survival Data
    a. Death Status
    b. Date of Death / Censoring Date
    c. Survival Time
10. Aggregated heath economics data recorded as an annual cost per patient for the following:
    a. Primary care prescriptions
    b. Primary care investigations
    c. Primary care consultations
    d. Primary care procedures and interventions
    e. Total primary care resource use

2. Data Items accessed by the research team, from LTHT's Databases and financial data derived data warehouse

From records for patients with the 20 most common cancers and a control cohort:

1. Demographics including
    a. age (<1 years and 1-4 years and 5-year bands thereafter, to 80+)
    b. Rurality score
    c. IMD quintile
    d. CCG
    e. sex
    f. ethnicity
    g. Weight and height
2. Date of cancer diagnosis or control patient hospital contact event
3. Cancer diagnosis (including primary and secondary cancer and recurrences) or clinical service of two-week wait or other urgent suspected cancer referral or control patient hospital contact event including
    a. National standard coded data, e.g. 3-digit ICD code
    b. Stage at presentation
    a. Morphology
    b. Grade
    c. Molecular phenotype e.g. ER/PR, HER2, EGFR
4. The presence/absence of specific co-morbidities (based largely on ACE-27, the 27-item comorbidity index for patients with cancer (HSCIC data dictionary, 2016) at or prior to diagnosis/control patient hospital contact event including:
    a. Myocardial Infarction
    b. Angina / Coronary Artery Disease

c. Congestive Heart Failure
d. Cardiac arrhythmia
e. Peripheral Arterial Disease
f. Hypertension
g. Venous disease including DVT, PE
    i. Venous Thromboembolic Disease
    ii. Venous Insufficiency
    iii. Varicose veins
h. Restrictive lung disease or COPD
i. Asthma
j. Liver Disease including chronic hepatitis
k. Stomach / Intestine including ulcers, malabsorption and inflammatory bowel disease
    iv. Peptic Ulcer Disease
    v. Malabsorption
    vi. Inflammatory Bowel Disease
l. Pancreatitis
m. Renal Disease
    vii. Grade 1
    viii. Grade 2
    ix. Grade 3
    x. Grade 4
    xi. Grade 5 (End Stage)
n. Diabetes mellitus
    xii. Type 1
    xiii. Type 2
    xiv. Other or Unspecified
    xv. Insulin dependent
    xvi. Diabetes Medication excluding insulin
    xvii. No Medication
o. Cerebral Vascular Accident
    xviii. Stroke
    xix. TIA
p. Paralysis including hemiplegia, paraplegia
    xx. Paralysis
    xxi. Cord injury and myelopathy
q. Neuromuscular
    xxii. Demyelinating disease
    xxiii. Parkinson's Disease
    xxiv. Motor Neurone Disease
r. Dementia
s. Mental Health Disorders
    xxv. Anxiety
    xxvi. Depression
    xxvii. Substance abuse including alcohol and illicit drug use
    xxviii. Post-Traumatic Stress Disorder
    xxix. Mania/Bipolar
    xxx. Behavioural Disorder
    xxxi. Schizophrenia / Psychosis
    xxxii. Perinatal Mental Health Disorder
    xxxiii. Obsessive Compulsive disorder
    xxxiv. Somatisation / Dissociation
    xxxv. Eating Disorder
    xxxvi. Attention Deficit and hyperactivity Disorder
t. Rheumatologic including rheumatoid, connective tissue disorder

xxxvii. Rheumatoid Arthritis
xxxviii. Psoriatic Arthritis
xxxix. Ankylosing Spondylitis
xl. Gout
xli. Back pain

u. HIV status
v. Obesity
w. Hyperlipidaemia
d. Women's Health Outcome measures

i. Assisted delivery
ii. Caesarean Section
iii. Cerebral venous sinus thrombosis
iv. Cervical dysplasia
v. Ectopic pregnancy
vi. Genital prolapse
vii. Infertility
viii. Maternal death
ix. Menopause
x. Menstrual Disorders
xi. Miscarriage
xii. Molar pregnancy
xiii. Multiple pregnancy
xiv. Normal delivery
xv. Painful intercourse
xvi. Post-natal depression
xvii. Precocious puberty
xviii. Pregnancy
xix. Psychosexual dysfunction
xx. Sterilisation
xxi. Stillbirth
xxii. Urogenital Fistula
xxiii. Combined oral contraception
xxiv. Contraceptive diaphragm
xxv. Contraceptive implant
xxvi. Contraceptive injectable
xxvii. Contraceptive patch
xxviii. Emergency Hormonal Contraception
xxix. IUD
xxx. IUD/IUS Removal
xxxi. IUS
xxxii. Progestogen only oral contraception
xxxiii. Removal of Contraceptive Implant

5. Development of specific co-morbidities (as above) annually for 10 years and 5-yearly intervals thereafter following cancer diagnosis/control patient hospital contact event - for comparison with the recording of these in primary care
6. Time since original diagnosis of specific co-morbidities and categories (as above) measured in 3 monthly increments in the year prior to cancer diagnosis (or control patient hospital contact event) and annually if longer than one year prior to diagnosis.
7. Dated information on cancer treatment received for primary diagnosis including surgery, chemotherapy, radiotherapy, hormone therapy, other
8. Dates and sites of cancer recurrence including

   a. National standard coded data, e.g. 3-digit ICD code
   b. Stage at presentation
   x. Morphology
   y. Grade
   z. Molecular phenotype e.g. ER/PR, HER2, EGFR
9. Survival data
   a. Death Status
   a. Date of death / Censoring Date
   b. Survival Time
   c. Cause of death
      i. 1a
      ii. 1b
      iii. 1c
      iv. 2
10. Hospital treatment received including surgery, chemotherapy, radiotherapy, hormone therapy at times of cancer recurrence
11. Dated information on results of radiological, pathological and biochemical results after cancer diagnosis or hospital contact event
12. Dated information on use of hospital resource over time including
   a. out-patient reviews by oncology doctor, nurse, AHP
   b. out-patient reviews by other doctor, nurse, AHP
   c. acute and elective admissions (number and duration) under oncology
   d. acute and elective admissions (number and duration) under other specialty
   e. number of diagnostic tests including blood, imaging, pathology, diagnostic scope annually for 10 years following cancer diagnosis/referral an subsequent recurrence events
   f. costs of treatment for cancer and subsequent care (late effects) derived from:
      i. LTHT's Patient Level Information and Costing System (PLICS)
      ii. Finance datasets used for DoH submissions for reimbursement under the national tariff system
      iii. LTHT income received (cost of care to the commissioners).
13. Summary data on diagnostic or severity scoring investigations for the comorbidities listed above:
   a. Most abnormal result in the 5 years before cancer diagnosis or control patient hospital contact event
   b. Most abnormal result in the 5 years after cancer diagnosis or control patient hospital contact event
   c. Mean, median, variance, interquartile range, minimum and maximum result annually from the 5 years prior to 5 years after cancer diagnosis or control patient hospital contact event
   d. Number of abnormal results in the 5 years before and 5 years after cancer diagnosis or control patient hospital contact event
14. **LTHT's derived Frailty scores:**
   a. **Frailty scores 5 years prior to cancer diagnosis or control patient hospital contact event.**
   b. **Month, year and new score for subsequent changes in Frailty score**

3. Data handling by University of Leeds authorised data managers

University of Leeds approved data managers will receive the data (that has gone through pseudonymisation procedures) required from LTHT and TPP to make the above fields available to researchers. The data management team will perform second-stage pseudonymisation:

1. Pseudonymise the patient record pseudonym using OpenPseudonymiser and a project-specific salt only known to the data management team (see the attached Data Flow Protocol for details about this process)
2. Aggregate dated codes or events to provide the research team with the binary (yes/no) indicators listed above (for example, presence or absence of cancer or the listed co-morbidities at annual stages, and eFI at annual stages)
3. Aggregate dated codes or events to provide the research team with counts within specific timeframes as listed above (for example, count of consultations per year following cancer diagnosis / urgent referral)
4. Replace month and year of birth with the age-bands listed above, based on age on date of cancer diagnosis / urgent referral.

The data management team will use automated procedures to do this, rather than manual data handling.
